# DNA Barcoding of Potential Mosquito Disease Vectors (Diptera, Culicidae) in Jazan Region, Saudi Arabia

**DOI:** 10.3390/pathogens11050486

**Published:** 2022-04-19

**Authors:** Elsiddig Noureldin, Denise Tan, Ommer Daffalla, Hatim Almutairi, Jaber Ghzwani, Majhalia Torno, Omar Mashi, Yahya Hobani, Huicong Ding, Abdullah Alamri, Khalid Shrwani, Ahmed Albarrag, Zaki Eisa

**Affiliations:** 1 Saudi Public Health Authority, Vector-Borne Diseases Laboratory, Jazan 45142, Saudi Arabia; ombakheit@cdc.gov.sa (O.D.); hatim.almutairi@hotmail.com (H.A.); jmghazwani@cdc.gov.sa (J.G.); osmashi@cdc.gov.sa (O.M.); yahobani@cdc.gov.sa (Y.H.); aialamri@cdc.gov.sa (A.A.); khalid_jebril@hotmail.com (K.S.); ambarraq@cdc.gov.sa (A.A.); zmomar@cdc.gov.sa (Z.E.); 2 Environmental Health Institute, National Environment Agency, 11 Biopolis Way, #06-05-08, Helios Block, Singapore 128667, Singapore; denise_tan2@rp.edu.sg (D.T.); majhalia_torno@nea.gov.sg (M.T.); ding_huicong@nea.gov.sg (H.D.); 3 School of Applied Science, Republic Polytechnic, 9 Woodlands Avenue 9, Singapore 738964, Singapore; 4 Department of Clinical Infection, Microbiology, and Immunology, School of Medicine, University of Liverpool, Liverpool L693BX, UK; 5 Department of Pathology, School of Medicine, King Saud University, Riyadh 11461, Saudi Arabia

**Keywords:** DNA barcode, mosquito taxonomy, phylogeny, Jazan, Saudi Arabia

## Abstract

The conventional morphological characterization of mosquito species remains heavily used for species identification in Jazan, Saudi Arabia. It requires substantial expertise and time, as well as having difficulty in confirming identity of morphologically similar species. Therefore, to establish a reliable and accurate identification system that can be applied to understanding spatial distribution of local mosquito species from the Jazan region, DNA barcoding was explored as an integrated tool for mosquito species identification. In this study, 44 adult mosquito specimens were analyzed, which contain 16 species belong to three genera of potential mosquito disease vectors (*Aedes*, *Anopheles*, and *Culex*). The specimens were collected from the Jazan region located in southwest Saudi Arabia. These included old and preserved mosquito voucher specimens. In addition, we assessed the genetic distance based on the generated mitochondrial partial *COI* DNA barcodes to detect cryptic diversity across these taxa. Nine mosquito species belonging to three genera were successfully barcoded and submitted to GenBank, namely: *Aedes aegypti*, *Aedes caspius*, *Aedes vexans*, *Aedes vittatus*, *Anopheles arabiensis*, *Culex pipiens*, *Culex quinquefasciatus*, *Culex sitiens*, and *Culex tritaeniorhynchus*. Of these nine species, *Aedes vexans*, *Aedes vittatus*, *Culex sitiens*, and *Culex tritaeniorhynchus* were registered in GenBank for the first time from Saudi Arabia. The DNA barcodes generated a 100% match to known barcodes of these mosquito species, that also matched with the morphological identification. *Ae. vexans* was found to be either a case of cryptic species (subspecies) or a new species from the region. However, more research has to be conducted to prove the latter. This study directly contributes to the development of a molecular reference library of mosquito species from the Jazan region and Saudi Arabia. The library is essential for confirmation of species in support of existing mosquito surveillance and control programmes.

## 1. Introduction

There are 112 genera and 3547 known species within the family Culicidae [1,2]. The most concerning of these are biting pests that transmit pathogens to humans and livestock [3]. The pathogens include viruses (dengue virus (DENV), Rift Valley fever virus and West Nile virus), protozoans (Plasmodium) (Marchiafava and Celli, 1885), and several nematodes [3,4]. They are also nuisance biters of humans and livestock that can strain valuable healthcare resources and loss in productivity [5,6,7].

To understand the dynamics of disease transmission and for the purpose of managing successful vector control programs, reliable and rapid identification of targeted mosquito species, together with the knowledge of their ecology and biology, are vital. Therefore, a critical first step would be to develop a species identification pipeline that is rigorous and can be implemented with minimal training.

Conventional morphological characterization of mosquito specimens remains heavily used for mosquito identification, even though it requires a specific substantial expertise and time [8]. In addition, available keys are quite specific. They are adapted for females, 3rd to 4th instar larvae, and specific countries or regions, which limits their application. Additionally, the method is limited to physically intact and/or preserved specimens, and is highly unreliable when handling polymorphic and cryptic species complexes [9,10].

Alternatively, molecular characterization or DNA barcoding is a widely accepted method for species identification which is efficient and precise [11]. This method enables researchers to identify mosquitoes up to subspecies level, help reconstruct their evolutionary histories and phylogenetic relationships, and understand genetic diversity amongst populations [11]. With their role as vectors in the transmission of pathogens of both medical and veterinary importance, mosquitoes are among the most intensely barcoded insect groups [12]. The number of studies on mosquito identification using DNA barcoding, based on a small region (658 bp) of the mitochondrial *cytochrome c oxidase subunit 1* (*CO1*) gene, has rapidly increased over the years [11,13]. Recent research showed that the *COI* DNA barcoding approach has been used as a molecular marker in identifying several mosquito species around the world [8,14,15,16,17,18,19].

The *COI* DNA barcoding method, however, does come with its own limitations. The approach has had limited success in identifying plant and fungi species [20,21,22]. It also failed to distinguish certain mosquito species, namely *Anopheles (such as Anopheles dacia and Anopheles messeae)*, *Aedes* *(Aedes sticticus*, *Aedes cantans*, *Aedes geminus*, *Aedes cinereus*, and *Aedes nigrinus*) *Culex* *(Culex pipiens* s.l.; *pipiens*, *molestus* and *quinquefasciatus*), *Culiseta* (*Culiseta fumipennis*, *Culiseta litorea*, and *Culiseta morsitans*), and two closely related species of *Ochlerotatus* [1,14,15,16,19,23,24]. In addition, the approach requires a comprehensive reference database for barcode comparison and matching for it to succeed [10]. Moreover, Duran et al. (2020) [25] found that tiger beetle species were frequently misidentified (24.5% of the time) when using COI barcodes, apparently due to mtDNA introgression amongst closely related species. It is worth noting that in animal mitochondrial gene trees, polyphyly is common and a taxonomically detected phenomenon [26]. The authors have significantly reviewed the major causes of mtDNA non-monophyly. Hence, an integrated approach for characterizing mosquitoes using both molecular and morphological identification is thought to be the most ideal for species identification [16].

To date, mosquito fauna of the Saudi Arabia comprises of 50 species belonging to seven genera and two subfamilies. The genus *Anopheles* (18 species) belongs to the subfamily Anophelinae, while *Aedes* (7 species), *Culex* (20 species), *Culiseta* (2 species), *Lutzia* (1 species), *Coquillettidia* (1 species), and *Uranotaenia* (1 species), belong to the subfamily Culicinae [27,28]. Culicinae is the largest mosquito group in Saudi Arabia, comprising 32 species (64%). However, there has been no formal surveys of genetic variation amongst mosquitoes found in the Jazan region, except for *Anopheles gambiae arabiensis*. Hence, there may be cryptic species yet to be discovered.

In the present study, DNA barcoding was used to complement morphological identification of 16 species belonging to three genera of potential mosquito disease vectors collected from the Jazan region, located in southwest Saudi Arabia. Some of the materials used are older (collected between 2009–2013), and all mosquito voucher specimens were dry preserved.

## 2. Materials and Methods

### 2.1. Study Area

**Figure 1:**
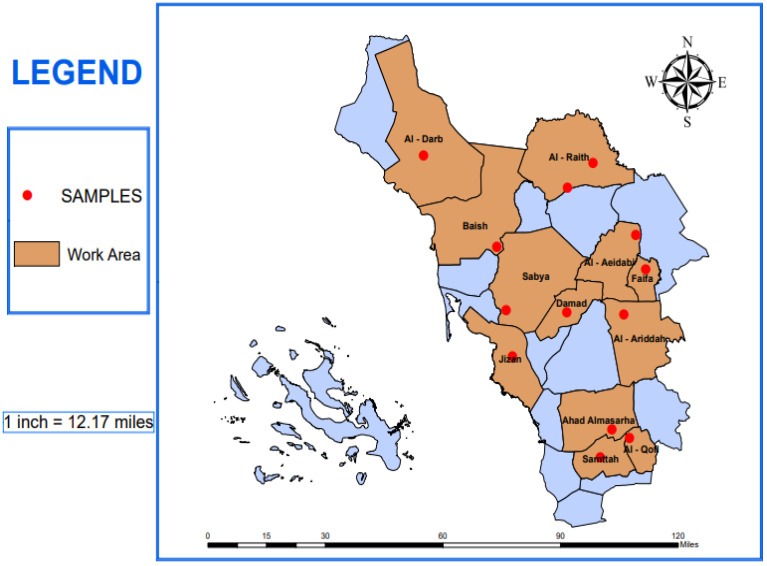
Boundaries of the different study areas in the Jazan region, southwest Saudi Arabia, showing the sites of mosquitoes’ collection (red dots).

Jazan has an area of about 22,000 km^2^, with a population of 1.6 million, that lies between 16°54′34.8588” N and 42°34′4.4472” E. It is located in the subtropical zone, south-west of Saudi Arabia. It is surrounded by the Red Sea from the west, the Arabic Republic of Yemen from the south and east, and the Asir region from the north. It has a coastal boundary of 250 km along the Red Sea and a 120 km border with the Republic of Yemen (Figure 1). The region includes over 3000 villages scattered throughout the area, and about 100 islands located in the Red Sea, including the Farasan islands. The topography of the area can be distinctly divided into three sectors: (a) the Sarwat Mountain range sector lies at the east (up to 2500 m above sea level (A. S. L.); (b) the hilly middle sector (300–600 m A.S.L.); and (c) Coastal sector lies at the west (30 m A.S.L.). The weather is subtropical, with an annual temperature around 35 °C, annual relative humidity ranging between 50–70%, and annual precipitation of 165 mm in the coastal sector, while it ranges between 300–500 mm in the Sarwat mountains ranges. [29,30]; GASTAT 2017: https://www.stats.gov.sa/en/5655. Accessed on 4 February 2021.

### 2.2. Mosquito Collection and Morphological Identification

CDC Miniature light traps were deployed for adult mosquito’ collection from different parts of the Jazan region from February 2018 to October 2019 (Table 1). Ten light traps were installed once per month in each of the houses and animals’ shelters in the vicinity of wild vegetation, near potential breeding sites (e.g., wadies (water streams), sewerage plants, dams, and ponds) from 1800–0600 hr. For outdoor collections, a 2-kg block of dry ice (CO_2_) was wrapped in a Hessian bag above the trap. To minimize mortality of the collected mosquitoes due to desiccation, damp cotton pads were kept in the collection cups. Collected mosquitoes were brought to the Vector-Borne Diseases Laboratory (VBDL) of the Saudi Public Health Authority (SPHA) in Gizan city for morphological identification. Taxonomic keys, as described in Bram (1967), Harbach (1985), Glick (1992), and Azari-Hamidian and Harbach (2009) [31,32,33,34], were used in mosquito species identification.

Larvae were collected by the dipping method during the routine mosquito field surveillance in the Jazan region. Field collected larvae were reared individually to adults. The adults were then identified by experienced taxonomists at the VBDL, Jazan, using the above-mentioned taxonomic keys. Each adult mosquito was assigned a reference name and number and then deposited as voucher specimens in the VBDL mosquito repository.

The morphologically identified adult mosquito species were then pinned following the method described by Gaffigan and Pecor (1997) [35], and shipped to the Environmental Health Institute (EHI), National Environment Agency (NEA) of Singapore for DNA barcoding. Some of old preserved mosquito voucher specimens from VBDL mosquito repository, namely: *Aedes vexans*, *Aedes vittatus*, *Anopheles dthali*, *Anopheles fluviatilis*, *Anopheles multicolor*, *Anopheles pretoriensis*, *Anopheles sergenti*, *Anopheles turkhudi*, *Culex quinquefasciatus*, and *Toxorhynchites* sp., were also sent for DNA barcoding.

### 2.3. DNA Extraction

Genomic DNA was extracted using two to three legs, from one side of the mosquito, in order to preserve the rest of the dried specimen for future reference. Where the specimen was damaged or incomplete, the entire thorax was used for extraction instead. The tissue was first homogenized (SPEX Sample Prep 1600 Mini G) and then digested overnight at 56 °C. DNA extraction was performed using the DNeasy Blood and Tissue Kit (Qiagen, Hilden, Germany), according to manufacturer’s specification. Total DNA was eluted into 100 µL buffer AE and stored at −20 °C.

### 2.4. Polymerase Chain Reaction (PCR) and Sequencing

A 709 bp fragment of the mitochondrial *cytochrome c oxidase subunit 1* gene (*COI*) was targeted for amplification using the following primer pair: COI_1490F 5′–TYT CAA CAA AYC AYA AAG AYA TTG G–3′ and COI_2198R 5′–TCW GGA TGH CCA AAR AAT CA–3′ (modified from Folmer et. al., 1994 [36]). Polymerase chain reactions were prepared in 20 μL reactions consisting of 10 µL 2X Phusion Flash PCR Master Mix (ThermoFisher Scientific, 168 Third Avenue. Waltham, MA, USA 02451), 1 µL of each primer (resulting in 0.5 µM final concentration), 4 µL template DNA and 4 µL H_2_O. The thermocycling profile was as follows: initial denaturation for 10 s at 98 °C, five cycles of 98 °C for 8 s, 50 °C for 15 s, and 72 °C for 30 s followed by 35 cycles of 98 °C for 8 s, 55 °C for 15 s, 72 °C for 30 s, and a final extension of 72 °C for 1 min. Amplified PCR amplicons were then examined on 1.5% agarose gels stained with GelRed (Biotium Inc., 46117 Landing Parkway Fremont, CA, USA). PCR purification and sequencing were performed by a commercial laboratory Axil Scientific, Singapore. All raw sequences were manually inspected and edited using Geneious v. 9.1.3 (Biomatters, Auckland, New Zealand). Multiple sequence alignment for PCR products was performed using the BioEdit program.

### 2.5. Phylogenetic Analysis

In total, 37 nucleotide sequences were used to construct the phylogeny. Each sequence pair had all ambiguous positions removed. In total, the final dataset contained 15,333 positions. Then, all sequences were aligned using MAFFT software with the default parameters [37]. Estimates of evolutionary divergence between sequences, and the number of base substitutions per site from between sequences were performed using the maximum composite likelihood model [38].

To ensure the accuracy of the phylogenetic reconstruction, preliminary optimization steps were performed, including estimating both the pairwise distance matrix and the best-fit substitution model using the MEGAX software [39].

The optimal substitution model was identified as the general time reversible model with gamma distribution rates (GTR + G), based on the lowest Bayesian information criterion (BIC) and Akaike information criterion (AIC) scores. The output of these optimization steps was used as input for reconstructing Bayesian phylogenetic trees using version 1.10.4 of BEAST software [40]. Prior to tree reconstruction, several assumptions were made as an input, including a constant population size, the use of the UPGMA tree as a starting point, and the use of the strict molecular clock, which assumes uniform rates across tree branches. The tree was then running over a period of ten million iterations, sampling every 1000th state and discarding the first 10%. The final tree was constructed from a consensus tree with a probability density of 95% (95%HPD) for each node. The tree figures were generated using FigTree software (FigTree ed.ac.uk. Accessed on 31 January 2022). All optimization data can be accessed via the Supplementary Material at https://doi.org/10.5281/zenodo.5901895. (Accessed on 31 January 2022).

**Figure 2:**
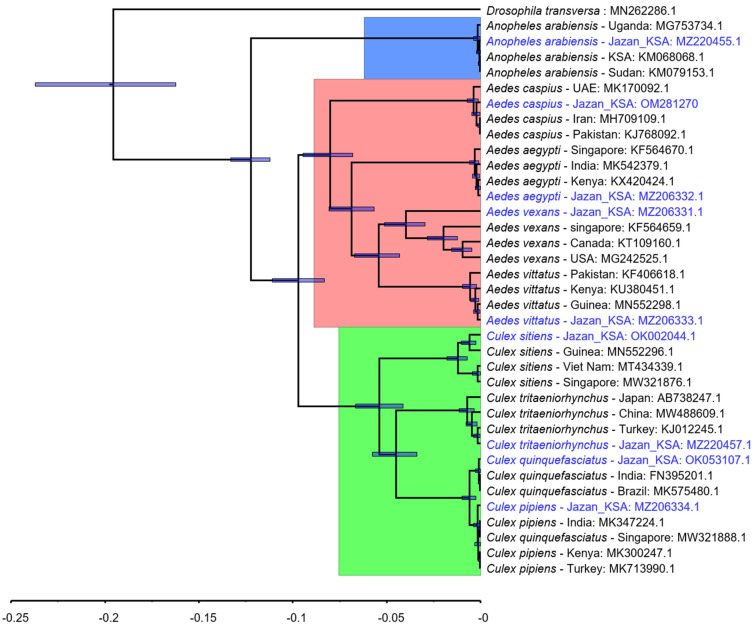
Bayesian phylogenetic tree of 37 *CO1* sequences samples in this study (including the one from Jazan region in blue) compared with other species from other different locations.

It is worth noting that there is more than one specimen for the same mosquito species with identical successful sequences (e.g., six *Aedes aegypti*, six *Aedes vexans*, two *Anopheles arabiensis*, nine *Culex sitiens*, five *Aedes caspius*, etc.—Table 2 and Table 3). The identical sequences for the mosquito species were only represented by one representative sequence in the phylogenetic tree (Figure 2).

## 3. Results

### 3.1. Mosquito Specimens’ Collection and Identification

In this study, 30,199 mosquitoes were collected throughout the course of the study from 12 governates of the Jazan region, southwest of Saudi Arabia (Figure 1, Table 1). Out of these, *Aedes aegypti* was the predominant species (55.9%), followed by *Culex quinquefasciatus* (22.4%), then *Culex* *tritaeniorhynchus* (8.2%).

A total of 81 mosquito specimens belonging to 17 species of four genera were analyzed. These include four species of *Aedes* (*n* = 35), four species of *Culex* (*n* = 26), eight species of *Anopheles* (*n* = 18), and two species of *Toxorhynchites* (*n* = 2), which were identified in this study (Table 2 and Table 3).

Out of the 81 specimens, 28 (34.6%) were collected as larvae and reared into adult before classification, 21 specimens (25.9%) were collected from the field as adults, and 32 (39.5%) were old specimens from VBDL mosquito repository.

Aedes species barcoded were: *Aedes aegypti*, *Aedes vexans*, *Aedes vittatus* and *Aedes caspius* (Table 2 and Table 3). Four species of Culex were barcoded: *Culex quinquefasciatus*, *Culex* *pipiens*, *Culex sitiens* and *Culex tritaeniorhynchus*. Likewise, there were eight species of Anopheles processed: *Anopheles gambiae arabiensis*, *Anopheles* *dthali*, *Anopheles fluviatilis*, *Anopheles multicolor*, *Anopheles pretoriensis*, *Anopheles sergenti*, *Anopheles stephensi* and *Anopheles turkhudi*. However, only *Anopheles gambiae arabiensis* was successful. Barcoding of Toxorhynchites was unsuccessful.

Sixty of the eighty-one specimens were analyzed at NEA (Singapore) (Table 2), while the remaining twenty-one were analyzed at the VBDL (Jazan, Saudi Arabia) (Table 3). The total successfully analyzed and sequenced mosquito specimens were 44 (54.3%).

Focusing on the successfully sequenced specimens, nine sequenced species belonging to three genera were registered in the GenBank, namely: *Aedes aegypti* (Linnaeus, 1762) MZ206332, *Aedes caspius* (Pallas, 1771) OM281270, *Aedes vexans* (Meigen, 1830) MZ206331, *Aedes vittatus* (Bigot, 1861) MZ206333, *Anopheles gambiae arabiensis* (Patton, 1905) MZ220455, *Culex pipiens* (Linnaeus, 1758) MZ206334, *Culex sitiens* (Wiedemann, 1828) OK002044, *Culex tritaeniorhynchus* (Giles, 1901) MZ220457, and *Culex quinquefasciatus* (Say, 1823) OK053107 (Table 4). Of these, four species: *Aedes vexans*, *Aedes vittatus*, *Culex sitiens* and *Culex tritaeniorhynchus* were molecularly identified and registered in the GenBank for the first time from Saudi Arabia. While another four species, *Aedes aegypti*, *Aedes caspius*, *Culex quinquefasciatus*, and *Culex pipiens* were registered in the GenBank for the first time from the Jazan region.

### 3.2. CO1 Based DNA Barcoding and Phylogenetic Tree

**Figure 3:**
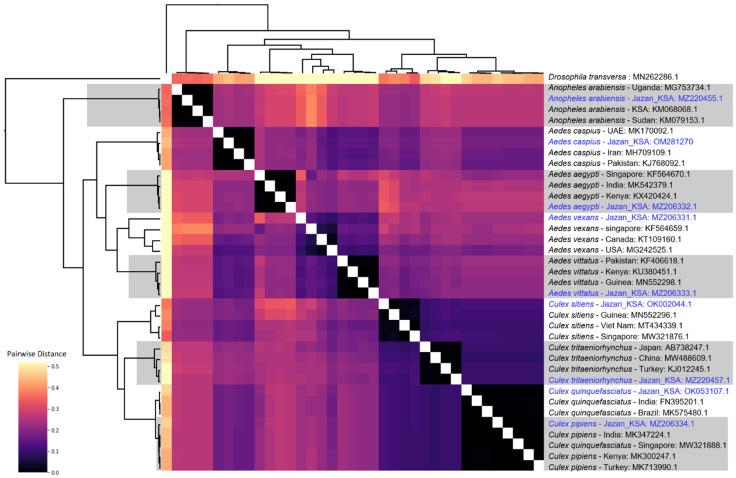
Estimates of evolutionary divergence between sequences. The number of base substitutions per site from between sequences are shown (our sequences from Jazan are colored in blue). Analyses were conducted using the maximum composite likelihood model [38]. The rate variation among sites was modeled with a gamma distribution (shape parameter = 0.84). This analysis involved 37 nucleotide sequences. All ambiguous positions were removed for each sequence pair (pairwise deletion option). There was a total of 15,333 positions in the final dataset. Evolutionary analyses were conducted in MEGA X [39].

The phylogenetic tree revealed distinct clustering for each species in the dataset, regardless of whether it was Jazan barcode DNA sequences or other similar barcode DNA sequences (Figure 2, Table 4). This distinct separation in the tree topology was achieved using Bayesian inference rather than neighbor-joining, maximum-likelihood, or minimum evolution trees (more details are available in the additional files at (https://doi.org/10.5281/zenodo.5901895. Accessed on 31 January 2022). Both previously published trees [41] and the pairwise distance matrix generated from the same dataset confirmed the tree topology (Figure 3). Given that bootstrapping has been criticized as biased in the genetics literature [42], it was not necessary because the tree was iterated ten million times, and the best consensus tree was constructed, with 95% confidence limits, as a result. Hence, we were able to compare the similarity between the sequences of our barcoded mosquitoes and the sequences of previously identified mosquitoes. This, in turn, verified the morphological identification of the specimens.

## 4. Discussion

Accurate identification of mosquito species is extremely important in vector surveillance and control programmes to detect mosquito species that play an important role in disease transmission. The recent advancement in DNA barcoding molecular techniques makes it possible to complement the morphological identification of mosquito species. However, careful morphological examination of mosquito species in combination with the application of molecular techniques should be made for a reliable identification [19].

In this study, some of the mosquito specimens that were processed for PCR produced no bands or faint ones (Table 2). This could be due to the condition of mosquito sample DNA which might have been degraded by oxidation and heat [43,44], or fumigation gas [45]. This suggests that, for the best results, DNA barcoding should be applied to fresh specimens or samples preserved in ideal preservation conditions for molecular work, viz., stored in ethanol, acetone, or refrigerated [10].

It appears from this and other research articles that some mosquito species cannot be successfully barcoded, presumably because intra-species genetic variations in the *CO1* gene are too great. Several investigators have discussed the matter in more detail, and came up with prominent explanations such as the mtDNA introgression amongst closely related species [25], polyphyly detection [26], genetic variation among congeners and conspecifics [46], and underestimating the rate of paraphyly due to operational factors and sampling effects [47]. Mutanen et al., (2016) [48] pointed out that misidentification, overlooked and over-splitting of species, and inherent subjectivity of species delimitations are among the factors that affect the non-monophyly in trees based on mitochondrial DNA.

The phylogenetic inferences based on the partial *COI* gene in this study showed that all mosquitoes clustered according to the related species or species complex that they were identified morphologically (Figure 2 and Figure 3). This demonstrates that *CO1* barcoding complements morphological identification, and the integration of both methods can be a useful tool for mosquito identification.

Our results showed that the *Aedes vittatus* sequences formed a monophyletic clade more than 99% of the time with those of Kenya (99.7%), Guinea (99.5%) and nearly 99% (98.9%) with Turkish species, suggesting that they are highly similar to each other (Table 4). Similarly, the *Aedes aegypti* sequences also formed a monophyletic group with GenBank *Ae. aegypti* sequence (KF564670) of Singapore (99.12%), and 99.5% with the Indian and Kenyan species (Table 4), indicating that the morphological identification of the *Ae. aegypti* samples in this study is highly accurate.

In the Jazan region, Kingdom of Saudi Arabia, *Aedes vittatus* is of concern due to its potential as a vector of pathogens posing a possible threat to human and animal health. The mosquito plays an important role in the transmission and maintenance of yellow fever (YFV) in some African countries, beside chikungunya (CHIKV), dengue (DENV) and Zika (ZIKV) viruses throughout its native range in Africa and Europe [49]. On the other hand, *Aedes aegypti* is widespread throughout the Jazan region, as well as the western region of the Kingdom of Saudi Arabia. Known as the primary dengue vector in Saudi Arabia [50], the species is of great public health importance in the Jazan region, and accurate identification is of utmost importance.

Focusing on the *Culex* genera, the *CO1* sequences of *Culex tritaeniorhynchus* were found to be closely related to species collected from Turkey, Japan, and China (99.54%, 99.08%, and 99.08%, respectively—Table 4). This is based on the constructed phylogenetic trees (Figure 2). Among the *Culex* species collected in this study, *Culex tritaeniorhynchus* comprised 8.2% of the total mosquito collected from the Jazan region (Table 1). It was found in different types of breeding habitats including dams, water tanks, man-made pools, rock pools, turbid and organically rich pools, and rain pools. It is the primary vector of rift valley fever (RVF) virus in the Jazan region, preferring to bite humans and sheep [51]. This species also transmits Japanese B encephalitis in the oriental and Southeast Asia region [31,52]. Having this in mind, *Culex tritaeniorhynchus* may pose a future health threat for transmitting some encephalitis in the Jazan region.

The *CO1* sequences of *Culex sitiens* and its constructed phylogenetic trees revealed that it is in close similarity to related species from Guinea, Vietnam and Singapore (98.8%, 98.5%, and 98%, respectively—Table 4, Figure 2). The species has been reported from the Jazan region by several authors [30,53,54,55], and is an implicated vector of Japanese B encephalitis [56]. Notably, Noureldin et al., (2021) [53] have recently used the *COI*-based molecular characterization to complement the morphological identification for *Culex tritaeniorhynchus*, *Culex quinquefasciatus*, *Culex pipiens*, and *Culex sitiens* in the Jazan region for the first time. Our analysis further supports the work of Noureldin et al. (2021) [53], providing more evidence that DNA barcoding is comparable to morphological identification of *Culex tritaeniorhynchus* and *Culex sitiens*. This method can be an alternative to morphological identification, which has the potential to scale up vector surveillance capabilities.

In the present study, the six sequenced *Aedes vexans* specimens were found genetically similar to one another and formed 93.94%, 93.77%, and 93.47% to the *Ae. vexans* reference sequences of USA, Canada, and Turkey, respectively (Table 1 and Table 4).

Considering that a 2–3% inter-species “barcode gap” is commonly adopted by researchers to delineate species [57], our morphologically identified *Aedes vexans* specimens from the Jazan region may be related but very unlikely to be truly *Ae. vexans*. This could suggest that either it is a case of cryptic species (subspecies), or a new species from the region. However, more research has to be done to prove the latter.

In UK and Europe, *Aedes vexans* showed some genetic differentiation and have distinct genotypes, and as a result were separated into two groups [19,58]. It is of note that *Aedes vexans* was previously associated in the rift valley fever (RVF) outbreak in 2000 in the Jazan region [51]. This species was found in large numbers, with up to 0.9% of the population harboring RVF virus during the outbreak.

In this study, the *CO1* barcodes of *Aedes (Ochlerotatus) caspius* were found to be closely related to specimens from Iran, Pakistan, and UAE (99.54%, 99.54%, and 99.39%, respectively—Table 4).

*Aedes caspius* is a competent vector of RVF virus [59]. It is a known floodwater mosquito that tends to breed in hotter and drier regions. The species is mainly found in coastal areas [60]. In disease investigation, a rapid and accurate identification of target species is essential, particularly to detect potential cryptic species which may be involved in disease transmission and ultimately affects the efficacy of control measures [61,62].

The barcode sequences of *Culex* *pipiens* showed that all *Culex pipiens* specimen formed a monophyletic group and were identical (100%) to species from Turkey, India and Kenya. On the other hand, *Culex quinquefasciatus* specimens were also identical (100%) to species found in Brazil, Singapore and India. *Culex pipiens* and *Culex quinquefasciatus* are conspecific individuals that do not form a monophyletic cluster in a gene tree. Globally, *Culex quinquefasciatus* and *Culex pipiens*, are the main vectors of urban bancroftian filariasis caused by the parasite, *Wuchereria bancrofti*. The disease has been frequently reported from the south-western regions of Saudi Arabia [63].

Even though there are no reports of diseases transmitted by *Aedes (Ochlerotatus)* *caspius*, *Aedes vittatus* and *Culex sitiens* in the Jazan region and the Kingdom of Saudi Arabia, the species were analyzed for their potential to transmit diseases in the future brought on by human and animal movement. Though they currently do not transmit diseases, they are likely to continue to cause nuisance and irritation in different parts of the Jazan region and Saudi Arabia. Hence, knowledge on these species is very important for early risk assessment, mitigation and control.

DNA barcoding could be used in the instances where mosquito specimens are damaged and their characters are indistinguishable, and in the case of the presence of subspecies or/and cryptic species. It could be also utilized to distinguish similar species, or to differentiate species if their larval stages cannot be distinguished from each other [31,64].

Overall, our study established that both morphological characterization and molecular barcoding are critical for accurate identification of mosquitoes found in the Jazan region. As such, an integration of methods should be pursued for future research aimed at surveying mosquitoes and determining species distribution. Likewise, future selective pressure analysis is recommended, but with more data.

## 5. Conclusions

In the present study, 44 adult mosquito specimens belonging to 16 species and three genera of potential mosquito disease vectors from the Jazan region, southwest Saudi Arabia, have been successfully analyzed. Nine species were morphologically identified, confirmed by DNA barcoding, and registered in the GenBank, four of which have been registered in the GenBank for the first time from Saudi Arabia.

The integrated approach to identification using both morphological and molecular methods allow for the differentiation of morphologically similar species and the determination of phylogenetic relationships between geographically separate specimen belonging to closely related or the same species. It is then proposed to use a combination of both methods in the identification of the mosquito fauna of Saudi Arabia. The finding of this study also encourages continuous research in the family Culicidae for the species delineation and the detection of cryptic genetic diversity within species groups (in this study, *Ae. vexans* was found to be either a case of cryptic species (subspecies) or a new species from the region. However, more research has to be done to prove the latter).

Most importantly, this study directly contributes to the development of a molecular reference library of the mosquito fauna in the Jazan region and Saudi Arabia. The library will be of vital importance and particularly essential for supporting the existing mosquito’s surveillance and control programmes.

## Figures and Tables

**Table 1:** Mosquito species composition and density in 12 governates of the Jazan region.

Municipality	*Aedes*				*Culex*				*Anopheles*		Total
	*aegypti*	*caspius*	*vittatus*	*vexans*	*tritaeniorhynchus*	*quinquefasciatus*	*sitiens*	*pipiens*	*arabiensis*	*dthali*	
Jizan	3969	148	0	0	664	3894	1068	160	4	10	9917
Damad	801	0	0	0	53	0	204	1	0	0	1059
Sabya	1513	0	0	0	253	1681	505	379	11	5	4347
Ahad Almasarha	2564	1	4	0	0	203	10	8	15	11	2816
Samttah	1322	0	0	0	0	2	1	0	0	0	1325
Al-Ariddah	1361	0	12	102	960	462	8	92	14	2	3013
Aliedabi	203	0	16	0	0	52	0	0	0	0	271
Faifa	685	0	202	0	157	114	0	112	5	0	1275
Al-Qofl	2093	0	267	0	199	159	120	44	41	16	2939
Baish	1192	0	0	0	23	9	2	65	11	7	1309
Al-Darb	882	0	255	0	166	123	0	63	45	7	1541
Al-Raith	296	5	7	0	3	73	0	2	1	0	387
**Total** %	**16,881** (55.9%)	**154** (0.5%)	**763** (2.5%)	**102** (0.3%)	**2478** (8.2%)	**6772** (22.4%)	**1918** (6.4%)	**926** (3.1%)	**147** (0.5%)	**58** (0.2%)	**30,199** (100%)

**Table 2:** DNA extraction methods and sequencing of *CO1* DNA barcodes of mosquitoes from the Jazan region (processed at NEA—Singapore).

Identified Mosquito Species	No. of Specimens	Tissue Sampled	PCR	Sequencing	Remarks
*Aedes aegypti*	6	2–3 legs	Success	Success	Adult reared from larvae in the lab
*Aedes vexans*	2	2–3 legs	Success	Success	Adult collected from the field
*Aedes vexans*	3	Head + thorax	Success	Success	Adult collected from the field
*Aedes vexans*	1	Whole mosquito	Success	Success	Adult reared from larvae in the lab
*Aedes vexans*	1	Whole mosquito	Success	Fail	Adult reared from larvae in the lab
*Aedes vexans*	4	Head + thorax	Faint band	-	Old preserved specimen
*Aedes vexans*	6	2–3 legs	No bands	-	Old preserved specimen
*Aedes vexans*	2	Head + thorax	No bands	-	Old preserved specimen
*Aedes vexans*	2	Whole mosquito	No bands	-	Old preserved specimen
*Aedes vittatus*	1	2–3 legs	Success	Success	Adult reared from larvae in the lab
*Aedes vittatus*	1	Whole mosquito	Success	Success	Adult reared from larvae in the lab
*Aedes vittatus*	1	2–3 legs	No bands	-	Old preserved specimen
*Anopheles arabiensis*	2	Whole mosquito	Success	Success	Adult reared from larvae in the lab
*Anopheles arabiensis*	2	Whole mosquito	Success	Fail	Adult reared from larvae in the lab
*Anopheles arabiensis*	4	2–3 legs	No bands	-	Old preserved specimen
*Anopheles dthali*	2	2–3 legs	No bands	-	Old preserved specimen
*Anopheles fluviatilis*	1	Head + thorax	No bands	-	Old preserved specimen
*Anopheles multicolor*	1	2–3 legs	No bands	-	Old preserved specimen
*Anopheles multicolor*	1	Whole mosquito	No bands	-	Old preserved specimen
*Anopheles pretoriensis*	2	Whole mosquito	No bands	-	Old preserved specimen
*Anopheles sergenti*	1	Thorax	No bands	-	Old preserved specimen
*Anopheles stephensi*	1	2–3 legs	No bands	-	Old preserved specimen
*Anopheles turkhudi*	1	Whole mosquito	No bands	-	Old preserved specimen
*Culex pipiens*	2	2–3 legs	Success	Success	Adult reared from larvae in the lab
*Culex pipiens*	3	Whole mosquito	Success	Success	Adult reared from larvae in the lab
*Culex quinquefasciatus*	2	2–3 legs	Success	Success	Adult reared from larvae in the lab
*Culex quinquefasciatus*	2	2–3 legs	Double bands	-	Adult reared from larvae in the lab
*Culex quinquefasciatus*	1	Whole mosquito	No bands	-	Old preserved specimen
*Toxorhynchites* spp.	2	2–3 legs	No bands	-	Old preserved specimen
**Total**	**60**	**-**	**-**	**23**	

**Table 3:** DNA extraction methods and sequencing of *CO1* DNA barcodes of mosquitoes collected from the Jazan region (processed at VBDL–Saudi Arabia).

Identified Mosquito Species	No. of Specimens	Tissue Sampled	PCR	Sequencing	Remarks
*Aedes caspius*	5	Whole mosquito	Success	Success	Adult reared from larvae in the lab
*Culex sitiens*	9	Whole mosquito	Success	Success	Adult collected from the field
*Culex* *tritaeniorhynchus*	7	Whole mosquito	Success	Success	Adult collected from the field
**Total**	**21**	**-**	**-**	**21**	

**Table 4:** GenBank accession numbers for sequences of the potential vector mosquitoes of the Jazan region, southwest Saudi Arabia, along with the closest available published sequence matches.

Species	GenBank Accession	Closest Available Published Sequence Matches
*Aedes aegypti*	MZ206332	99.55% *Ae.* *aegypti* MK542379 India 99.54% *Ae.* *aegypti* KX420424 Kenya 99.12% *Ae.* *aegypti* KF564670 Singapore
*Aedes caspius*	OM281270	99.54% *Ae. caspius* MH709109 Iran 99.54% *Ae. caspius* KJ768092 Pakistan 99.39% *Ae. caspius* MK170092 UAE
*Aedes vexans*	MZ206331	93.94% *Ae.* *vexans* MG242525 USA 93.77% *Ae.* *vexans* KJ208504 Canada 93.47% *Ae.* *vexans* MF095664 Turkey
*Aedes vittatus*	MZ206333	99.70% *Ae.* *vittatus* KU380451 Kenya 99.54% *Ae.* *vittatus* MN552298 Guinea 98.93% *Ae.* *vittatus* KF406618 Pakistan
*Culex pipiens*	MZ206334	100% *Cx.* *pipiens* MK713990 Turkey 100% *Cx.* *pipiens* MK347224 India 100% *Cx.* *pipiens* MK300247 Kenya
*Culex quinquefasciatus*	OK053107	100% *Cx. quinquefasciatus* MK575480 Brazil 100% *Cx. quinquefasciatus* MW321888 Singapore 100% *Cx. quinquefasciatus* FN395201 India
*Culex sitiens*	OK002044	98.77% *Culex sitiens* MN552296 Guinea 98.52% MT434339 Viet Nam 98.00% *Culex sitiens* MW321876 Singapore
*Culex tritaeniorhynchus*	MZ220457	99.54% *Cx. tritaeniorhynchus* KJ012245 Turkey 99.08% *Cx. tritaeniorhynchus* AB738247 Japan 99.08% *Cx. tritaeniorhynchus* MW488859 China
*Anopheles gambaie arabiensis*	MZ220455	100% *An. arabiensis* KM068068 Saudi Arabia 100% *An. arabiensis* KM079153 Sudan 99.85% *An. gambiae* MG753743 Uganda
*Drosophila transversa*	MN262286	Outgroup

## Data Availability

All data presented in this study are available in the main text, figures, tables and Supplementary Materials.

## Supplementary Materials

The following are available online (Phylogenetic analysis: optimization and tree construction) at https://doi.org/10.5281/zenodo.5901895: Figure2.BEAST. Annotated trees: BEAST final annotated tree in NEXUS format; Figure2.BEAST. beauti: BEAUTI input file, Figure2.BEAST.log: BEAST log file for the 10 million iterations; Figure2. BEAST. ops: BEAST operator analysis; Figure2.BEAST. trees: BEAST trees files generated after 10 million iterations; Figure2.BEAST.xml: BEAST input file in XML format; Figure2.MAFFT. fasta: extracted sequences after aligned using MAFFT; Figure2.fasta: tree input sequences extracted from NCBI database; Figure2.png: Figure2 tree in PNG image format; Figure2.tree: Figure2 tree in NEXUS format; Figure3.Matrix.csv: pairwise distance matrix saved in CSV file format; Figure3.png: Figure3, Gamma_Parameter.txt: maximum likelihood estimates of gamma parameter for site rates generated with MEGAX, and best-fit-Model.txt: table of maximum likelihood fits of 24 different nucleotide substitution models generated with MEGAX.

## Abbreviations

SPHA	Saudi Public Health Authority
VBDL	Vector-Borne Diseases Laboratory
NEA	National Environment Agency
EHI	Environmental Health Institute
*CO1*	Cytochrome c oxidase subunit 1
DNA	Deoxyribonucleic acid
BIC	Bayesian information criterion
AIC	Akaike information criterion
UAE	United Arab Emirates
